# A Genome-Scale DNA Repair RNAi Screen Identifies SPG48 as a Novel Gene Associated with Hereditary Spastic Paraplegia

**DOI:** 10.1371/journal.pbio.1000408

**Published:** 2010-06-29

**Authors:** Mikołaj Słabicki, Mirko Theis, Dragomir B. Krastev, Sergey Samsonov, Emeline Mundwiller, Magno Junqueira, Maciej Paszkowski-Rogacz, Joan Teyra, Anne-Kristin Heninger, Ina Poser, Fabienne Prieur, Jérémy Truchetto, Christian Confavreux, Cécilia Marelli, Alexandra Durr, Jean Philippe Camdessanche, Alexis Brice, Andrej Shevchenko, M. Teresa Pisabarro, Giovanni Stevanin, Frank Buchholz

**Affiliations:** 1 Max Planck Institute for Molecular Cell Biology and Genetics, Dresden, Germany; 2 Structural Bioinformatics, BIOTEC TU, Dresden, Germany; 3 INSERM, Unit 975 Paris, France; 4 Université Pierre et Marie Curie-Paris6, Centre de Recherche de l'Institut du Cerveau et de la Moelle Epinière, Paris, France; 5 CNRS, Unité Mixte de Recherche 7225 Paris, France; 6 Hôpital Nord, Saint Etienne, France; 7 Hôpital Neurologique, Lyon, France; 8 APHP, Pitié-Salpêtrière Hospital, Department of Genetics and Cytogenetics, Paris, France; Medical Research Council Human Genetics Unit, United Kingdom

## Abstract

DNA repair is essential to maintain genome integrity, and genes with roles in DNA repair are frequently mutated in a variety of human diseases. Repair via homologous recombination typically restores the original DNA sequence without introducing mutations, and a number of genes that are required for homologous recombination DNA double-strand break repair (HR-DSBR) have been identified. However, a systematic analysis of this important DNA repair pathway in mammalian cells has not been reported. Here, we describe a genome-scale endoribonuclease-prepared short interfering RNA (esiRNA) screen for genes involved in DNA double strand break repair. We report 61 genes that influenced the frequency of HR-DSBR and characterize in detail one of the genes that decreased the frequency of HR-DSBR. We show that the gene KIAA0415 encodes a putative helicase that interacts with SPG11 and SPG15, two proteins mutated in hereditary spastic paraplegia (HSP). We identify mutations in HSP patients, discovering KIAA0415/SPG48 as a novel HSP-associated gene, and show that a KIAA0415/SPG48 mutant cell line is more sensitive to DNA damaging drugs. We present the first genome-scale survey of HR-DSBR in mammalian cells providing a dataset that should accelerate the discovery of novel genes with roles in DNA repair and associated medical conditions. The discovery that proteins forming a novel protein complex are required for efficient HR-DSBR and are mutated in patients suffering from HSP suggests a link between HSP and DNA repair.

## Abstract 2

We have identified a novel gene in a genome-wide, double-strand break DNA repair RNAi screen and show that is involved in the neurological disease hereditary spastic paraplegia.

## Author Summary

All cells in our bodies have to cope with numerous lesions to their DNA. Cells use a battery of genes to repair DNA and maintain genome integrity. Given the importance of an intact genome, it is not surprising that genes with roles in DNA repair are mutated in many human diseases. Here, we present the results of a genome-scale DNA repair screen in human cells and discover 61 genes that have a potential role in this process. We studied in detail a previously uncharacterized gene (KIAA0415/SPG48) and demonstrated its importance for efficient DNA double strand break repair. Further analyses revealed mutations in the SPG48 gene in some patients with hereditary spastic paraplegia (HSP). We showed that SPG48 physically interacts with other HSP proteins and that patient cells are sensitive to DNA damaging drugs. Our data suggest a link between HSP and DNA repair and we propose that HSP patients should be screened for KIAA0415/SPG48 mutations in the future.

## Introduction

Mutations in DNA repair genes are associated with different diseases and disorders including cancer [1], accelerated aging [2], and neuronal degeneration [3]. Neurons appear to be particularly vulnerable to mutations in DNA repair genes, possibly due to the lack of proliferation and high oxidative stress within these cells. As a consequence, several neurological diseases have been linked to defects in DNA repair such as Ataxia-telangiectasia [4], Ataxia-telangiectasia-like disorder [5], Seckel syndrome [6], Nijmegen breakage syndrome [7], and Charcot-Marie-Tooth syndrome [8].

A particularly dangerous DNA lesion for a cell is a double strand break (DSB), in which two strands of the DNA are broken in close proximity to one another [9],[10]. DSBs are repaired mainly via two parallel pathways: homologous recombination and nonhomologous end joining (NHEJ). Repair via homologous recombination typically restores the genetic information, whereas repair via NHEJ often leads to mutations [10],[11].

Recently, several RNAi screens have addressed different aspects of mammalian DNA repair, such as increased sensitivity towards PARP inhibition [12], increased sensitivity towards cisplatin [13], accumulation of 53BP1 foci [14],[15], or altered phosphorylation of the histone variant H2AX [8]. These screens have greatly enhanced our understanding of human DNA repair processes and delivered a number of novel genes implicated in various aspects of DNA repair. Here, we report a genome-scale RNAi screen for genes implicated in homologous recombination-mediated DSB repair, uncovering a variety of known and so far uncharacterized genes implicated in this process. In this work, we mine this screen employing a structural bioinformatics approach and identify KIAA0415/SPG48 as a putative helicase that is associated with hereditary spastic paraplegia (HSP).

## Results

### Genome-Scale RNAi Screen

**Figure 1:**
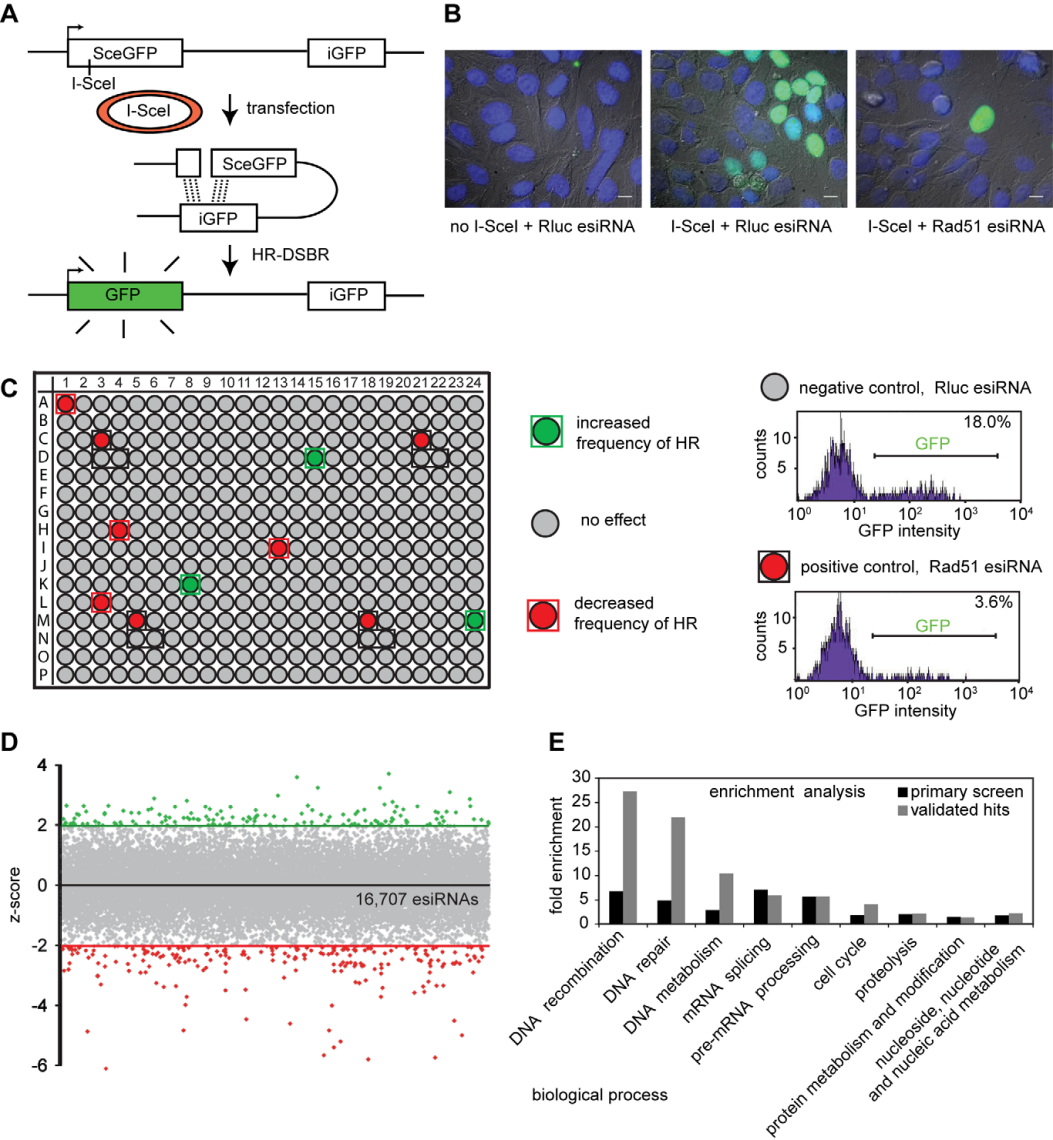
Genome-scale HR-DSBR esiRNA screen. (A) Schematic representation of the DR-GFP assay. The two non-functional GFP alleles and the I-SceI cutting site are shown. The transfected plasmid encoding the I-SceI endonuclease is presented as a red circle. The functional GFP gene that is generated after successful HR-DSBR is shown in green. (B) Immunofluorescence analysis of the DR-GFP HeLa cell line after transfection with or without the I-SceI endonuclease plasmid and indicated esiRNAs. Scale bars represent 10 µm. (C) Analysis of an example plate from the screen. Grey wells indicate knockdowns that did not significantly change the percentage of GFP positive cells observed, and red and green wells denote knockdowns that decreased or increased the percentages of GFP positive cells observed, respectively. Control wells are marked with black frames. On each plate there were four positive controls (esiRNA targeting Rad51) and eight negative controls (esiRNA against Rluc - renilla luciferase). Example FACS histograms for the control transfections are presented. (D) Dot plot of the primary screen. Results are presented as average z-scores derived from two independent replicates. Knockdowns with z-scores below −2 or above 2 are shown in red or green, respectively. (E) Results of the gene ontology enrichment analysis for the primary (black) and validated (grey) hits.

For a comprehensive search of genes associated with DNA DSB repair, we performed a genome-scale RNAi screen, utilizing an endoribonuclease-prepared short interfering RNA (esiRNA) library [16] and employing the well-established DR-GFP assay [17]. First, a stable HeLa cell line with two non-functional GFP alleles was generated, in which GFP expression is efficiently activated only after HR-DSBR (Figure 1A). We then tested the robustness of the assay by co-transfection of these cells with the I-SceI expression plasmid and an esiRNA targeting Rad51, which is an essential factor for the early stages of homologous pairing and strand exchange [18]. Depletion of Rad51 resulted in a marked reduction of GFP positive cells, and comparisons to negative control transfected cells suggested a high dynamic range for candidate factors influencing HR-DSBR (Figure 1B and histograms Figure 1C).

The RNAi screen was carried out in duplicate in 384-well plates by co-transfection of an I-SceI encoding plasmid with the individual esiRNAs targeting over 16,000 human genes [16]. The percentage of GFP positive cells was determined by high throughput FACS, providing a sensitive readout for esiRNAs influencing the frequency of HR-DSBR (Figure 1C). Knockdown of 228 and 141 transcripts significantly decreased or increased the percentage of GFP positive cells, respectively (Figure 1D, Table S1). Among the strongest knockdowns affecting HR-DSBR were genes with well-characterized roles in DNA repair such as Rad51, BRCA1, and SHFM1. Gene ontology enrichment analysis of the candidates revealed a 5-fold enrichment for genes reported to be implicated in DNA repair (Figure 1E), confirming that the screen was efficient.

### Hit Validation

To validate the candidate hits we examined their expression in HeLa cells and resynthesized all esiRNAs for the genes that were expressed. We also generated a second, independent, and non-overlapping esiRNA for these genes and tested all esiRNAs again in the DR-GFP assay in multiple replicates. Using stringent selection criteria (see Online Methods), 45 genes decreased the frequency of homologous recombination, while 17 genes increased it with two independent silencing triggers (Table 1). To further narrow down the list of these 62 candidates, we tested the esiRNAs for their impact on intracellular GFP levels. EsiRNAs that influence GFP levels, for example by targeting a transcriptional activator for GFP expression, could score in the DR-GFP assay and contaminate the hit list. We therefore transfected the esiRNAs into GFP expressing HeLa cells and assayed GFP levels by FACS. EsiRNAs targeting MKNK2 reduced GFP levels in these cells. Therefore, this gene was excluded from further analysis, reducing the final hit list to 61 genes (Table 1). The effectiveness of this stringent validation was monitored again by gene ontology enrichment analysis, with an enrichment of now 20-fold for genes annotated in the category DNA repair (Figure 1E).

**Table 1:** Summary of phenotypic data of the candidate genes implicated in HR-DSBR.

Gene Name	Ensembl ID	Frequency of HR-DSBR		GFP Levels	Viability	Increased Sensitivity			Gamma-H2AX Assay
		esiRNA 1	esiRNA 2			Cisplatin	MMC	IR	
ACTB	ENSG00000075624	↓	↓↓	−	−	−	−	−	−
AIP	ENSG00000110711	↓	↓	−	−	−	++	−	−
ALOX15	ENSG00000161905	↓↓	↓↓	−	+	−	++	−	−
ARHGEF1	ENSG00000076928	↓↓	↓↓	−	+	−	+	−	↓ 1h, ↓ 6h
BCAM	ENSG00000187244	↓↓	↓↓	−	+	−	+	−	−
BRCA1	ENSG00000012048	↓↓↓	↓↓↓	−	+	+	+++	+	↑ 6h
C1orf63	ENSG00000117616	↓↓	↓↓	−	−	+	+	+	↑ 6h
CHCHD2	ENSG00000106153	↓↓	↓↓	−	+	−	++	−	−
DRG2	ENSG00000108591	↓↓	↓↓	−	−	−	+++	−	−
ETFB	ENSG00000105379	↓↓	↓↓	−	+	−	++	−	−
FAM110C	ENSG00000184731	↓↓	↓	−	+	−	−	−	−
FIZ1	ENSG00000179943	↓↓	↓	−	−	−	+	−	↓ 6h
GAK	ENSG00000178950	↓↓	↓↓	−	−	−	+	−	−
HNRPA0	ENSG00000177733	↓↓	↓↓	−	−	−	+	−	↓ 6h
IGLON5	ENSG00000142549	↓↓	↓↓	−	−	−	+	−	−
KIAA0415	ENSG00000164917	↓↓	↓↓	−	−	−	++	−	−
NTHL1	ENSG00000065057	↓↓	↓↓	−	−	−	+++	−	↑ 6h
OSBPL5	ENSG00000021762	↓	↓↓	−	−	+	−	++	−
PRPF40B	ENSG00000110844	↓↓	↓↓	−	−	−	++	−	↑ 6h
PSMD4	ENSG00000159352	↓↓	↓↓↓	−	+	−	+	−	−
RAD51	ENSG00000051180	↓↓↓	↓↓↓	−	+	+++	+++	++	↑ 6h
RBBP8	ENSG00000101773	↓↓	↓↓	−	−	−	++	+	↑ 6h
RBM42	ENSG00000126254	↓↓	↓↓	−	−	−	++	−	−
RECQL4	ENSG00000160957	↓↓	↓	−	−	−	++	+	−
SEMA7A	ENSG00000138623	↓↓	↓↓	−	+	−	−	−	−
SERPINH1	ENSG00000149257	↓↓	↓	−	+	−	−	−	−
SHFM1	ENSG00000127922	↓↓↓	n.a.	−	−	++	+++	−	−
TRMT2A	ENSG00000099899	↓↓	↓	−	+	+	−	−	↑ 6h
TSKU	ENSG00000182704	↓	↓↓	−	−	−	+	−	−
XPC	ENSG00000154767	↓↓	↓↓	−	+	−	++	−	−
ZMYND15	ENSG00000141497	↓	↓↓	−	−	−	+++	−	−
ARCN1	ENSG00000095139	↓	↓↓	−	++	n.d	n.d	n.d	n.d
CKAP5	ENSG00000175216	↓↓	↓↓	−	++	n.d	n.d	n.d	n.d
CWC22	ENSG00000163510	↓↓↓	↓↓	−	++	n.d	n.d	n.d	n.d
DDB1	ENSG00000167986	↓↓	↓↓	−	++	n.d	n.d	n.d	n.d
E2F1	ENSG00000101412	↓	↓↓	−	++	n.d	n.d	n.d	n.d
HNRPK	ENSG00000165119	↓↓	↓↓	−	++	n.d	n.d	n.d	n.d
PSMD1	ENSG00000173692	↓↓↓	↓↓↓	−	++	n.d	n.d	n.d	n.d
PSMD14	ENSG00000115233	↓↓	↓↓↓	−	++	n.d	n.d	n.d	n.d
SNRNP200	ENSG00000144028	↓↓	↓↓↓	−	++	n.d	n.d	n.d	n.d
THOC4	ENSG00000183684	↓↓	↓↓	−	++	n.d	n.d	n.d	n.d
TPX2	ENSG00000088325	↓↓	↓↓↓	−	++	n.d	n.d	n.d	n.d
VPRBP	ENSG00000145041	↓↓↓	↓↓	−	++	n.d	n.d	n.d	n.d
ZYG11BL	ENSG00000160445	↓	↓↓	−	++	n.d	n.d	n.d	n.d
MKNK2	ENSG00000099875	↓↓	↓↓	+	−	n.d	n.d	n.d	n.d
ATXN3	ENSG00000066427	↑↑	↑↑	−	−	n.d	n.d	n.d	n.d
C5orf28	ENSG00000151881	↑↑	↑↑	−	−	n.d	n.d	n.d	n.d
C6	ENSG00000039537	↑↑	↑↑	−	−	n.d	n.d	n.d	n.d
CNGA1	ENSG00000198515	↑↑	↑↑	−	−	n.d	n.d	n.d	n.d
CREBBP	ENSG00000005339	↑	↑↑↑	−	−	n.d	n.d	n.d	n.d
DNAJB4	ENSG00000162616	↑↑	↑↑	−	−	n.d	n.d	n.d	n.d
LIG4	ENSG00000174405	↑↑	↑↑	−	−	n.d	n.d	n.d	n.d
LYRM7	ENSG00000186687	↑↑	↑↑	−	−	n.d	n.d	n.d	n.d
MMRN1	ENSG00000138722	↑↑	↑↑	−	−	n.d	n.d	n.d	n.d
PDHX	ENSG00000110435	↑↑	↑↑	−	−	n.d	n.d	n.d	n.d
SLC39A12	ENSG00000148482	↑	↑↑	−	−	n.d	n.d	n.d	n.d
SMCHD1	ENSG00000101596	↑↑↑	↑↑	−	−	n.d	n.d	n.d	n.d
SMS	ENSG00000102172	↑↑	↑↑	−	−	n.d	n.d	n.d	n.d
STAG2	ENSG00000101972	↑↑	↑↑	−	−	n.d	n.d	n.d	n.d
USP12	ENSG00000152484	↑↑↑	↑↑	−	−	n.d	n.d	n.d	n.d
WRB	ENSG00000182093	↑↑	↑↑	−	−	n.d	n.d	n.d	n.d
XRCC2	ENSG00000196584	↑↑	↑↑	−	−	n.d	n.d	n.d	n.d

Calculated z-scores using esiRNA against Rluc as negative controls are shown for two independent esiRNA and marked with arrows. Genes that after knockdown decreased the frequency of HR below z-score −4, −2, or −1.5 are marked with ↓↓↓, ↓↓ or ↓, respectively; genes that increase the frequency of HR over z-score 4, 2, or 1.5 are marked with ↑↑↑, ↑↑, or ↑, respectively; n.a., not available. Genes that decreased GFP levels with a z-score>4 are marked with +. Genes that after knockdown decreased viability below 50% and 25% are marked with ++ and +, respectively. Genes that after knockdown decrease the cell number when treated with cisplatin, MMC, or IR by 40%, 30%, or 10% in comparison to Rluc transfection are marked with +++, ++, and +, respectively; n.d., not done. In the last column arrows indicate ↑ increased and decreased ↓ number of gamma-H2AX positive cells after 1 h or 6 h post-IR for knockdowns statistically different (*p*<0.05) from Rluc transfections.

### Knockdowns That Increased the Frequency of HR-DSBR

Silencing of 17 genes significantly increased the number of GFP positive cells in the DR-GFP assay. Hence, the knockdown of these genes promoted HR-DSBR, which might be of interest for several biological applications such as increasing the targeting efficiency of genes by homologous recombination [19]. Different reasons might account for the increased number of GFP positive cells observed. One possibility is that the knockdown led to an inhibition of the NHEJ pathway, thereby shifting the ratio of the two possible pathways toward repair via HR. Support for this reasoning comes from experiments in yeast and flies, where the knockout of DNA ligase IV, a gene that is required for NHEJ [20], significantly increased gene targeting by homologous recombination [21],[22]. Interestingly, the knockdown of human Lig4 resulted in a striking increase in GFP positive cells in the DR-GFP assay (Table 1), suggesting that inhibition of the NHEJ pathway can increase the frequency of HR-DSBR also in mammalian cells. This idea is further supported by inspection of other known NHEJ proteins, including XRCC4, XRCC5, XRCC6, PRKDC, and DCLRE1C [23],[24]. Knockdown of all of these proteins increased the frequency of homologous recombination in the DR-GFP assay (Table S1). Hence, we speculate that other genes that increased the number of GFP positive cells might be implicated in the NHEJ pathway and that knockdown of these genes could enhance gene targeting by homologous recombination in mammalian cells.

### Knockdowns That Decreased the Frequency of HR-DSBR

The list of genes that decreased the frequency of HR-DSBR was enriched for proteins with well-defined roles in HR-DSBR, such as Rad51 and BRCA1. In addition, genes, such as E2F1, that more indirectly influence HR-DSBR were also identified in the screen. E2F1 is involved in cell cycle and apoptosis regulation after DNA damage [25] and has recently been implicated in transcriptional regulation of Rad51 and BRCA1 [26], possibly explaining why the knockdown of E2F1 scored in our screen. Interestingly, the assay also uncovered a number of genes that have roles in DNA repair processes other than HR-DSBR, such as XPC, which has a role in nucleotide excision repair (NER) [27], and the base excision repair (BER) DNA helicase RECQL4 [28]. However, a polymorphism in the XPC gene has recently been shown to correlate with bleomycin-induced chromosomal aberrations [29], and RECQL4 has been reported to coincide with foci formed by Rad51 after induction of DSBs [30], suggesting possible links between the different DNA repair pathways. Finally, the gene list is enriched for proteasome subunits, including PSMD4, PSMD1, PSMD14, and SHFM1. Treatment with proteasome inhibitors has been shown to specifically suppress HR-DSBR possibly because of the lack of proteasome-mediated degradation of chromatin bound proteins blocking the access to the lesion [31],[32]. Moreover, SHFM1 has been shown to be required for Rad51 foci formation upon DNA damage [33], implicating a more direct role of this proteasome subunit in HR-DSBR and possibly providing an explanation why SHFM1 was one of the strongest hits in our screen. Based on these results we were encouraged to investigate further the knockdowns that decreased the number of GFP positive cells in the DR-GFP assay.

**Figure 2:**
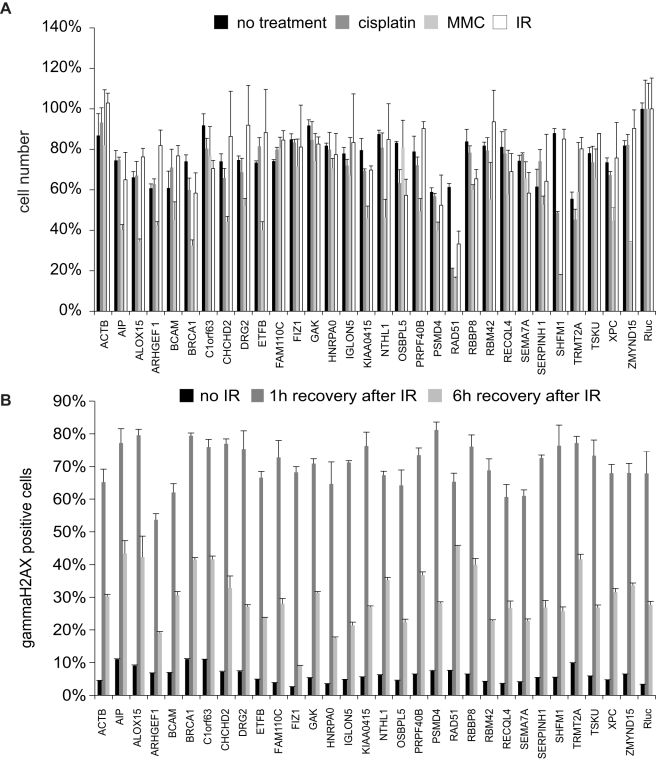
Secondary assays for genes that decrease the frequency of HR-DSBR. (A) Effect on the cell viability after transfection of indicated esiRNAs (black) in combination with cisplatin (dark grey), MMC (light grey), and IR (white) treatment are shown. Error bars indicate standard deviation. All results were normalized to esiRNA against Rluc transfections. (B) Effect on percent of gammaH2AX positive cells after transfection of indicated esiRNAs without irradiation (black), 1 h post-irradiation (dark grey), and 6 h post-irradiation (light grey). Error bars indicate standard deviation.

To characterize in detail the 44 knockdowns that decreased the frequency of HR-DSBR, we performed several additional assays. First, we tested the influence on cell viability of these esiRNAs in HeLa cells. Thirteen esiRNAs considerably decreased cell numbers and were excluded from further analyses (Table 1). Second, we performed mitomycin C (MMC), cisplatin, and ionizing radiation (IR) sensitivity assays. MMC predominantly causes interstrand cross-links, which result, among other things, in DSBs due to a block of replication forks [34]. Cisplatin damages DNA in a different way and generates predominantly intrastrand cross-links [35], whereas IR gives rise to a variety of DNA lesions [36]. Cells with impaired DNA repair pathways might be more sensitive to these treatments, which should manifest in reduced cell viability. Twenty-four hours post-transfection of the esiRNAs, the cells were treated for 1 h with MMC, cisplatin, or exposed to IR and cells were counted after an additional incubation for 48 h. A number of knockdowns increased the sensitivity towards one or more treatments, substantiating a role of these genes in DNA repair, with some of the knockdowns showing an effect for one, but not the other treatment (Figure 2A, Table 1). For instance, the knockdown of RBBP8 (also known as CtIP), which promotes DNA end resection [37], did not cause increased sensitivity towards cisplatin. However, substantially less cells were counted after MMC treatment, indicating that RBBP8 depletion primarily sensitized the cells against this drug. Third, we employed a gamma-H2AX removal assay. The histone H2AX is phosphorylated on serine 139 predominantly by ATM/ATR [38],[39] at sites of DSBs until the lesion is repaired. After successful DNA repair this phosphorylation is reverted by the phosphatase PP2A [40]. Several knockdowns resulted in extended time before gamma-H2AX was removed from irradiated cells (Figure 2B, Table 1), suggesting a delay in DSBR, and potentially explaining the observed reduction of GFP positive cells in the DR-GFP assay. Surprisingly, a few knockdowns showed overall reduced numbers of gamma-H2AX positive cells, or accelerated removal of gamma-H2AX after irradiation. For example, depletion of ARHGEF1 resulted in a reduced number of gamma-H2AX positive cells 1 h after irradiation (Figure 2B). Potentially, this Rho guanine nucleotide exchange factor [41] is required for efficient recruitment of H2AX phosphorylation factors, which ultimately translates into less efficient HR-DSBR. In contrast, the knockdown of FIZ1, a Flt3 interacting zinc finger protein [42], resulted in similar numbers of gamma-H2AX positive cells 1 h after irradiation in comparison to the control transfected cells. However, gamma-H2AX was more rapidly removed in these cells (Figure 2B), potentially compromising effective DSBR. Taken together, these results validate the effectiveness of our screen and serve as an initial classification of molecular pathways for a number of genes that can be explored in future studies.

### KIAA0415 Is a Putative Helicase Required for Efficient HR-DSBR

**Figure 3:**
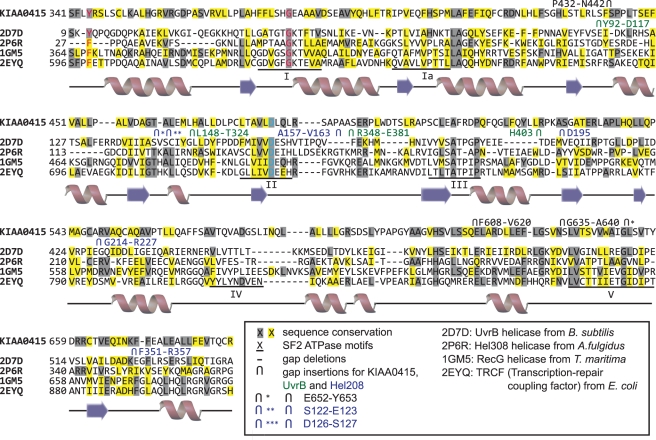
Structure-based sequence alignment of the helicase C domains of the SF2 helicases UvrB (2D7D), Hel308 (2P6R), RecG (1GM5), and TRCF (2EYQ). Their consensus secondary structure elements are shown bellow as red spirals (α-helices) and blue arrows (β-strands). The sequence alignment of KIAA0415 obtained from threading and used to build a 3D model of its putative helicase C-like domain based on these structural templates is shown at the top. Sequence conservation of KIAA0415 with respect to the template structures is highlighted in grey (conservative) and yellow (semi-conservative). Gap deletions and insertions are represented by dashed lines and inverted U symbols, respectively. Insertions are labelled with the corresponding N- and C-ending residue numbering (black for KIAA0415, green for UvrB, and blue for Hel208). Regions I, Ia, II, III, IV, and V of consensus SF2 helicase motifs are underlined. Residues involved in ADP- and Mg^2+^ binding are coloured in blue and red, respectively.

For this work, we mined the screen by performing bioinformatics analyses on the uncharacterized sequences in an attempt to reveal possible molecular functions. KIAA0415 emerged as particularly notable. By applying threading techniques (see Online Methods for details), we identified potential structural homologies of KIAA0415 with proteins belonging to the fold family “P-loop containing nucleoside triphosphate hydrolases” (SCOP c.37; Table S2). This fold family contains the so-called “helicase C domain” (PF00271) formed by a tandem repeat of two RecA-like domains (Tandem AAA-ATPase superfamily). Top scoring sequence-to-structure alignments were obtained with the KIAA0415 sequence and the structure of the helicase C domains of SF2 helicases that are involved in DNA repair such as UvrB, Hel308, RecG, and TRCF (Figure 3). Visual inspection of the generated 3D model (see Online Methods) confirmed the existence of potential SF2 helicase motifs in KIAA0415 (Figure S1). Molecular dynamics simulations were used to refine the KIAA0415 model and corroborated its stability and its putative ADP and Mg^2+^ recognition (Video S1, Online Methods). These results further support the prediction of a helicase-like domain within KIAA0415 and substantiate the conservation in 3D of residues important for its function as a putative SF2 helicase.

**Figure 4:**
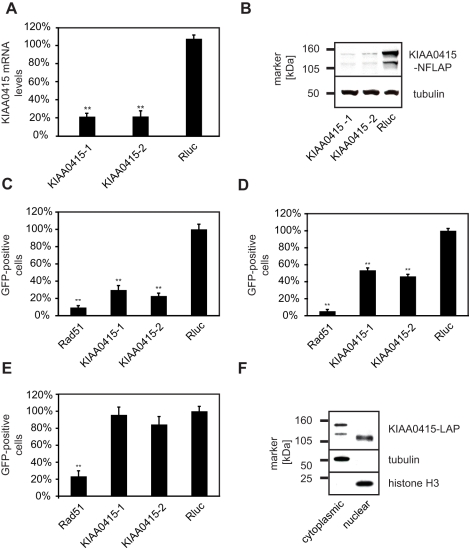
Functional analysis of KIAA0415. (A) Efficiency of KIAA0415 mRNA knockdown with two independent esiRNAs in HeLa cells. Relative levels of mRNA 24 h post-transfection of indicated esiRNAs are shown. Error bars indicate standard deviation, **p<0.01. (B) Efficiency of KIAA0415 protein knockdown with two independent esiRNAs in HeLa cells. KIAA0415-LAP Western blot analysis of HeLa cell extracts 48 h post-transfection of indicated esiRNAs is shown. A blot against tubulin served as the loading control. (C) Two independent KIAA0415 esiRNAs influence the frequency of homologous recombination repair measured utilizing the DR-GFP assay. The relative percentage of GFP positive HeLa cells, normalized to the Rluc transfected cells, is shown for indicated esiRNAs. Error bars indicate standard deviation. **p<0.01. (D) The KIAA0415 knockdown phenotype is not cell line dependent. The relative percentage of GFP positive U2OS cells, normalized to the Rluc transfected cells, is shown for indicated esiRNAs. Error bars indicate standard deviation, **p<0.01. (E) Expression of the mouse KIAA0415 orthologue rescues the KIAA0415 RNAi phenotype. The relative percentage of GFP positive in DR-GFP HeLa cells stably expressing the mouse KIAA0415 from a BAC transfected with indicated esiRNAs are shown. Error bars indicate standard deviation, **p<0.01. (F) Different KIAA0415 isoforms are found in the nucleus and in the cytoplasm. A KIAA0415 Western blot of HeLa cell extracts after cell fractionation is shown. Blots against tubulin and histone H3 served as controls.

Based on these results, we decided to further elucidate possible molecular functions of KIAA0415. We first tested the potency of the employed KIAA0415 esiRNAs in more detail. Both esiRNAs efficiently depleted KIAA0415 mRNA transcripts (Figure 4A) and protein (Figure 4B). We then repeated the DR-GFP assay in the HeLa reporter cell line and found 3.4 (esiRNA1) and 4.3 (esiRNA2) fold decrease in GFP positive cells in comparison to controls, suggesting reduced frequencies of homologous recombination (Figure 4C). We examined the expression levels of I-SceI after the knockdowns to rule out the possibility that I-SceI-generated DSBs are compromised (Figure S2). To exclude a possible cell-type specific effect, we also tested the knockdowns in a different cell line. U2OS cells carrying a single insertion site of the DR-GFP construct showed a similar reduction of GFP positive cells upon KIAA0415 knockdown (Figure 4D), indicating that this effect was not cell line specific. Finally, we excluded possible off-target effects by performing cross-species RNAi rescue experiments [43]. Stable expression of mouse KIAA0415 in the human DR-GFP cell line rendered this cell line resistant to the human esiRNAs, authenticating a role of KIAA0415 in HR-DSBR (Figure 4E). In summary, these results suggest that KIAA0415 is a novel putative SF2 helicase required for efficient HR-DSBR.

### KIAA0415 Forms a Complex with Proteins Associated with Spastic Paraplegia

**Figure 5:**
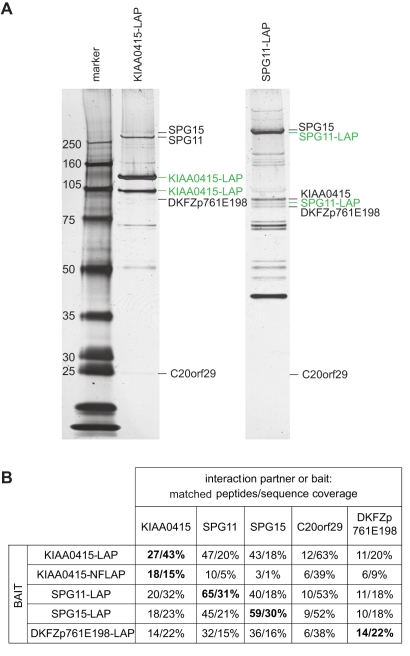
KIAA0415 interacts with SPG11, SPG15, DKFZp761E198 and C20orf29. (A) SDS-PAGE gels obtained from the immunoprecipitation of KIAA0415-LAP and SPG11-LAP. Baits (marked in green) and prey (marked in black) were identified by in-gel digestion and nanoLC-MS/MS analysis (see Figure S3). Bands that are not marked represent unspecific background proteins or bait specific proteins (see Table S3, and Online Methods). (B) The composition of KIAA0415 protein complex analyzed as established by shotgun-LC-MS/MS (see Table S3). The number of matched detected peptides and protein sequence coverage are shown. Results for bait proteins are marked in bold.

**Figure 6:**
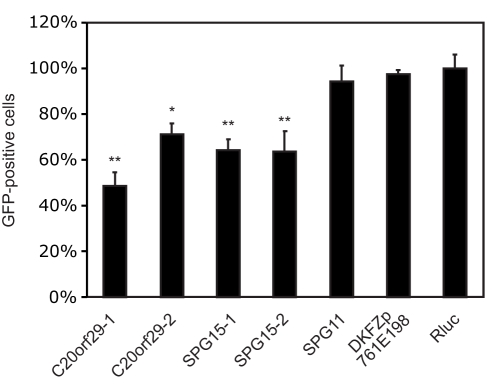
KIAA0415 interactors are required for efficient HR-DSBR. The relative percentage of GFP positive HeLa cells, normalized to the Rluc transfected cells, is shown for indicated esiRNAs. Error bars indicate standard deviation, *p<0.05, **p<0.01.

To further characterize KIAA0415, we tagged the gene on a bacterial artificial chromosome (BAC) applying the TransgeneOmics approach [44]. This method allows expression of tagged proteins from its native promoter in its genomic context, and therefore, the protein is expressed near physiological levels [44],[45]. C- and N-terminally tagged KIAA0415 was successfully cloned and expressed in HeLa cells. The fusion protein showed disperse, cytoplasmic, and nuclear localization, which did not change considerably upon induction of DNA damage (unpublished data). Immunoblotting of cell extracts revealed two major protein bands, possibly reflecting two KIAA0415 isoforms (Figure 4B). Cell fractionations showed that the shorter isoform was predominantly nuclear, whereas the longer form was found mostly in the cytoplasm (Figure 4F). Immunoprecipitation experiments followed by spectrometric identification of co-isolated proteins revealed interactions of KIAA0415-LAP with SPG11, SPG15, C20orf29, and DKFZp761E198 (Figure 5A,B and Table S3). In order to validate these interactions we generated cell lines expressing C-terminally tagged SPG11, SPG15, and DKFZp761E198 again using the TransgeneOmics approach. Reciprocal immunoprecipitation experiments followed by mass spectrometry analyses of in-gel and in-solution digests confirmed the existence of a protein complex, which consists of at least five core proteins: KIAA0415, SPG11, SPG15, C20orf29, and DKFZp761E198 (Figure 5B and Table S3). In order to test whether protein interaction partners of KIAA0415 would also affect HR-DSBR, we tested esiRNAs targeting these genes in the DR-GFP assay. Interestingly, significant reduction of GFP positive cells were observed upon silencing of C20orf29 and SPG15 with two independent esiRNAs (Figure 6), suggesting that these proteins are also required for efficient HR-DSBR. Knockdown of SPG11 and DKFZp761E198, however, did no have an effect on the percentage of GFP positive cells. Together, these experiments reveal a novel protein complex, which at least in part is required for efficient HR-DSBR.

### KIAA0415 Is Mutated in Patients with Spastic Paraplegia

The KIAA0415 interaction partners SPG11 and SPG15, also known as spatacsin and spastizin, are encoded by two genes that have been associated with hereditary spastic paraplegia with thin corpus callosum (HSP-TCC) [46],[47]. HSP-TCC is a subset of hereditary spastic paraplegia (HSP), which are inherited neurological disorders caused by the degeneration of the cortico-spinal tracts leading to lower-limb spasticity. HSP is a highly heterogeneous condition with at least 46 loci identified so far [48]. A potential interaction of SPG11 and SPG15 has been suggested on the basis of similar neurological symptoms [49], however a physical interaction of SPG11 and SPG15 has not been reported thus far. Because of the physical interaction of KIAA0415 with these two proteins encoded by genes associated with HSP, we decided to investigate if any unexplained HSP cases could be linked to mutations in KIAA0415. Direct sequencing of KIAA0415 in 166 unrelated HSP patients, including 38 and 64 cases with a recessive or dominant inheritance pattern and 64 sporadic cases (see Online Methods), identified 7 known and 15 new variants, respectively. Most of these variants were not considered causative, because they did not affect protein sequence, were not predicted to alter correct splicing, or were also found frequently in control samples (Table S4). However, one of these identified variants led to a premature stop codon at position 527 (c.1413_1426del14/p.L471LfsX56, Table S4) and was absent in 158 Caucasian and 84 North-African control chromosomes. The mutation was heterozygous and no other mutation or variant was found in the coding sequence of KIAA0415 or in its regulatory regions in this apparently sporadic patient (FSP-70-1). No other subjects from the family were available for sampling and no copy number variations were detected on chromosome 7 in the affected patient (unpublished data), but small heterozygous rearrangements or mutations in uncovered regions (unknown exons or introns) might have escaped detection.

**Figure 7:**
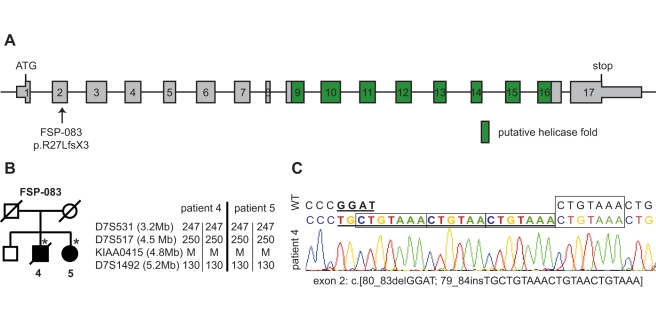
KIAA0415 mutation in HSP patients. (A) Schematic representation of the KIAA0415 exon structure. The location of the homozygous mutation and the predicted putative helicase fold (in green) are indicated. (B) Pedigree of KIAA0415 mutation in the FSP-083 family. Square symbols represent men; the circles represent women. Symbols of dead subjects are crossed. The filled symbols indicate affected individuals. The numbers below individuals are an internal reference for each sampled individual. Stars indicate sampled subjects. Segregation of the mutation and of 3 microsatellites markers on chromosome 7p are shown next to the pedigree, relative to their position in Mbases. The alleles of the microsatellites are given in base pairs; M, mutation. (C) Electropherograms of KIAA0415 mutations. Deviating sequences from the wt sequence are underlined.

More interestingly, we also found a homozygous mutation in two French siblings (FSP-083), which was not detected in 156 Caucasian and 242 North-African control chromosomes. In these patients, a complex indel in exon 2 (c.[80_83del4;79_84ins22], Figure 7C) generates a frameshift and a stop codon following amino-acid 29 (p.R27LfsX3, Figure 7A). Interestingly, the insertion is an imperfect quadruplication of the sequence CTGTAA(A), suggesting DNA polymerase slippage during DNA synthesis as the mechanism for introduction of the mutation. Both affected patients presented with progressive spastic paraplegia associated with urinary incontinence since age 50 and 49, respectively. Cerebral MRI was normal but spinal hyperintensities at C3-C4 and C7 were observed in one. Both parents died at the age of 72 and 77, respectively, of non neurological causes. They originated from two neighbouring villages, but there was no known consanguinity. However, the analysis of three close microsatellite markers (D7S531, D7S517, and D7S1492) and the loss of heterozygosity (LOH) search using CYTO_12 microarrays (unpublished data) confirmed that the region is homozygous in both affected patients (Figure 7B).

**Figure 8:**
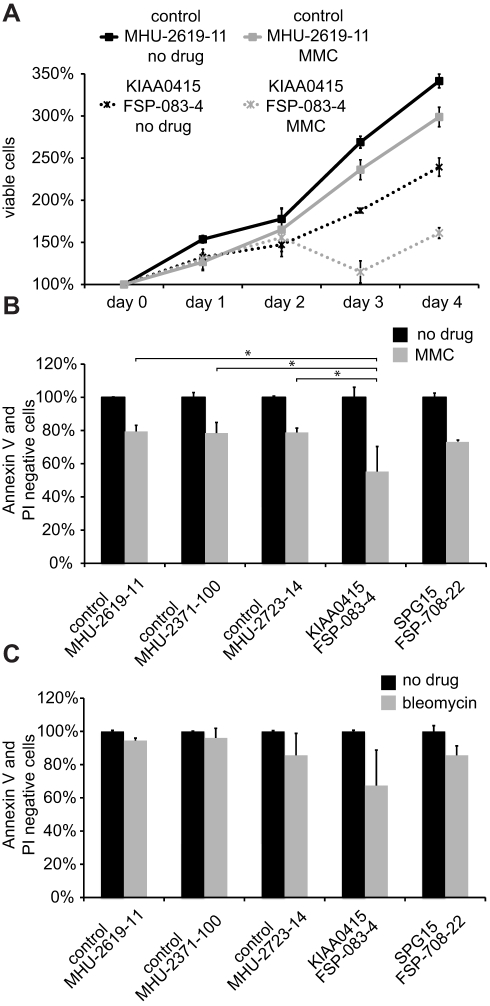
Sensitivity of lymphoblast cell lines to DNA damaging agents. (A) Growth curves of a control lymphoblast cell line—MHU-2619 (solid line), a cell line carrying the KIAA0415 mutation—FSP-083-4 (dotted line) without drug treatment (black line) or MMC treatment (light grey), are presented. (B and C) Cell lines derived from patients with a mutation in KIAA0415 and SPG15 are more sensitive to DNA damaging drugs. The decrease of viable cells (propidium iodide and Annexin V negative) is shown in percent (grey bar) for the indicated cell lines after MMC treatment (B) and bleomycin treatment (C). *p<0.05.

To further substantiate a role of KIAA0415 in DNA repair, we compared drug sensitivity in lymphoblast cell lines established from a patient carrying the KIAA0415 mutation (FSP-083-4) and a patient carrying a mutation in SPG15 (FSP-708-22 [50]) to control lymphoblast cell lines. Strikingly, the KIAA0415 mutant cells were significantly (*p*<0.05) more sensitive to MMC and bleomycin treatments compared to any of the control cell lines (Figure 8). In addition, also the SPG15 cell line showed a mild sensitivity to these drugs, phenocopying the results observed in HeLa and U2OS cells.

Taken together, these experiments identify KIAA0415 as a novel gene, which is mutated in patients with HSP, and implicate a link between HSP and DNA repair.

## Discussion

Using a well-characterized esiRNA library [16] we performed a genome-scale RNAi screen and identified 61 genes that reproducibly decreased or increased the frequency of DNA repair in an assay for homologous recombination [17]. Secondary assays for processes relevant to DNA repair corroborated many of the initial findings. Hence, we provide a dataset that should accelerate the discovery of novel genes with roles in DNA repair and associated medical conditions. Eighteen out of the 61 candidate genes have been described in other large-scale mammalian DNA repair studies [8],[13],[15],[51], demonstrating the effectiveness of our screen, but also highlighting that the use of different assays can uncover novel players. Hence, we predict that the development of alternative DNA repair assays for RNAi screens will reveal additional genes implicated in DNA repair. For our screen we co-transfected the “DNA damaging reagent,” I-SceI, together with the esiRNA silencing triggers. Hence, proteins with long half-lives may have been missed in this screen. Assays in which the DSB is introduced some time after the cells were transfected with the silencing triggers could uncover additional genes playing a role during DNA repair.

To prioritize the molecular investigation of the uncharacterized proteins identified in the screen, we employed a structural bioinformatics approach. Based on the prediction that KIAA0415 represents a novel putative helicase we investigated this gene in more detail. Tagging of the gene using the TransgeneOmics approach revealed nuclear as well as cytoplasmic localization and physical interaction with at least four proteins. Investigations of the interaction partners showed that at least two of these proteins are also required for efficient HR-DSBR. Possibly, these proteins form a complex that is required for efficient HR-DSBR. Consequently, the complex would lose its activity when one of the three proteins is depleted.

Two of the interaction partners of KIAA0415 are encoded by genes that are associated with spastic paraplegia. This result prompted us to examine whether KIAA0415 mutations can explain spasticity in patient samples not linked with mutations in any of the known spastic paraplegia genes. We report a homozygous mutation in KIAA0415, responsible for the spastic paraplegia observed in two siblings. Hence, we identify KIAA0415 as a novel spastic paraplegia associated gene. Based on this finding, we propose to rename KIAA0415 to SPG48 according to the HUGO nomenclature. The fact that three proteins that form a protein complex result in similar phenotypic consequences argues that the whole complex is exerting an important function, which is disturbed when one of the proteins is missing or non-functional. It will therefore be interesting to investigate the remaining interaction partners, C20orf29 and DKFZp761E198, for possible mutations in HSP patients, even though they do not map to known HSP loci [49]. Although only demonstrated for one case, cell lines derived from a patient carrying a SPG48 mutation were more sensitive to DNA damaging drugs than control cells, corroborating a role of SPG48 in DNA repair. Unfortunately, material from other patients with SPG48 mutations was not available. However, we propose that in the future HSP patients be screened for mutations in SPG48 and that cells from these individuals be checked for DNA repair defects.

Genes mutated in HSP have been associated with several biological functions, including intracellular transport, axonal pathfinding, mitochondrial functions, cholesterol metabolism, myelin formation/stability, and chaperonin activity [48]. Based on our findings, we propose that HSP might also be a result of impaired DNA repair, adding HSP to the growing list of neurodegenerative diseases caused by DNA repair deficiencies [4],[5],[7],[8]. In agreement with this hypothesis, SPG11 has recently been reported to be phosphorylated upon DNA damage by ATM or ATR [51]. Whether SPG48 (and its associated proteins) is a direct component of the HR-DSBR pathway or more indirectly linked to DNA repair remains to be established. Biochemical analysis of the putative helicase domain of SPG48 appears to be an attractive entry point into gaining mechanistic insights into the DNA repair function(s) of SPG48.

The technological advances in RNAi screening have increased the speed at which phenotypic data can be obtained. However, interpretation of the resulting genotype-phenotype relationships remains challenging, and approaches that help to decipher the screening data are highly desirable. Approaches that analyze phenotypic data from unrelated RNAi screens and that combine phenotypic- with localization- and proteomic data [52],[53] have been used successfully to bootstrap phenotype-to-function analyses. Here, we explored the possibility of combining RNAi screening data with structural bioinformatics approaches. The obtained results demonstrate that this combination generates valuable information, which helps to prioritize the follow-up studies of uncharacterized candidate genes. We envision that the design of an automatic pipeline to analyze possible structural and functional features beyond protein sequence similarities will further accelerate the characterization of genes identified in RNAi screens. In the future, it will be important to combine the different “omics” and bioinformatics approaches to understand DNA repair at a systems level and to further accelerate the discovery of genes relevant to human pathology.

## Materials and Methods

### Generation of HeLa DR-GFP Cell Lines

Ten µg of the DR-GFP construct [17] were transfected into 2.5×10^6^ HeLa cells using 12 µl Enhancer (Qiagen) and 14 µl Effectene (Qiagen) according to the manufacturer's protocol. Stable cell lines were selected with 3 µg/ml puromycin (Sigma-Aldrich) and single clones were obtained by FACS sorting on a FACSAria (BD Biosciences). Colonies derived from individual clones were expanded and tested for their behaviour after transfection with a plasmid encoding the I-SceI endonuclease. A cell line with virtually no GFP positive cells before I-SceI treatment and high number of GFP positive cells after I-SceI treatment was chosen for the screen.

### Immunofluorescence Microscopy Analysis

Cells were grown on glass coverslips and fixed with 3% paraformaldehyde (PFA) as described previously [44]. Immunofluorescence stainings were performed with a primary mouse anti-GFP antibody (Roche Diagnostics, 1∶4,000 dilution) and a secondary donkey anti-mouse antibody conjugated to Alexa488 (Molecular Probes, 1∶500 dilution). Genomic DNA was counterstained with ProLong Gold antifade reagent containing DAPI (Invitrogen). Images were acquired on an Axioplan II Microscope (Zeiss) operated through MetaMorph (Molecular Devices).

### Western Blot Analysis

Western blot analysis was performed as described previously [52]. In this study the following primary antibodies were used: mouse anti-GFP (Roche Diagnostics, 1∶4,000 dilution), mouse anti-DM1alpha tubulin (MPI-CBG Antibody Facility, 1∶50,000 dilution), and rabbit anti-Histone H3 (Abcam 1∶25,000 dilution).

### Genome-Scale esiRNA Screen

The esiRNA library employed has been described elsewhere [16],[54]. For the screen the I-SceI expression plasmid [17] was co-transfected with individual esiRNAs in an arrayed fashion. Briefly, 50 ng of each esiRNA in 5 µl TE Buffer was pipetted into 384-well tissue culture plates (BD Biosciences) and stored at −20°C. Each plate contained four esiRNAs against Rad51 as positive control (at positions C3, C21, M5, M18) and 12 esiRNAs targeting renilla luciferase (Rluc) as negative control (at positions C4, D3, D4, C22, D21, D22, M6, N5, N6, M19, N18, N19 as shown in Figure 1C). Using a multi-well dispenser (WellMate, Thermo Scientific) a mixture of the I-SceI plasmid (12.75 ng/well) and the Enhancer (0.142 µl/well) in 5 µl/well EC Buffer (Qiagen) was dispensed and briefly spun in a Heraeus Multifuge 4KR (Thermo Electron Corporation). After incubation for 5 min, Effectene (0.12 µl/well) diluted in 5 µl/well EC Buffer was added to each well and plates were briefly spun again. The transfection mixture was incubated for 5 min to allow complex formation. In the meantime HeLa cells carrying the DR-GFP reporter construct were harvested, counted, and diluted to a final concentration of 40 cells/µl in DMEM (Invitrogen) containing 12.5% Fetal Bovine Serum (Invitrogen). Fifty µl of the cell suspension was added to each well using a multi-well dispenser (Wellmate, Thermo Scientific). In order to prevent evaporation, plates were sealed with breathable plate sealing foils (Corning) and incubated in a tissue culture incubator at 37°C in 5% CO_2_. The medium was replaced 24 h post-transfection. After another 72 h cells were washed with PBS and detached by adding 15 µl/well trypsin/EDTA (Invitrogen). After 25 min cells were fixed by addition of 15 µl/well 3% PFA and stored no longer than 48 h at 4°C. Cells were assayed with a FACSCalibur (BD Biosciences) equipped with a High Throughput Sampler (BD Biosciences). Data were acquired and analyzed using CellQuest Pro (BD Biosciences).

### Hit Evaluation

Z-scores were calculated for the percentages of GFP positive cells using the following equation: z = (x−μ) σ^−1^ with: x − percentage of GFP positive cells; μ − mean percentage of GFP positive cells; σ − standard deviation of the number of GFP positive cells. In the primary screen mean and standard deviations were calculated separately for each plate over all samples on the plate excluding controls. Z-scores were calculated for each esiRNA and averaged for duplicates. The transfection of esiRNA targeting Rad51 was used as positive control and as reference for the assay performance. esiRNAs for which the average z-score was below −2 or over 2 were considered as primary hits (Table S1).

In further validation experiments, the z-scores were calculated based on the mean and standard deviation of the negative control (Rluc transfection). EsiRNAs for which the average z-score for 4 replicates were below −2 or over 2 for one esiRNA and below −1.5 or over 1.5 for a second esiRNA were classified as validated hits. Primer sequences for utilized esiRNAs are presented in Table S5.

Gene enrichment analysis was performed using the Panther Analysis Tools (http://www.pantherdb.org/tools/).

### Cisplatin/MMC/IR Sensitivity Assay

Fifteen ng of each esiRNA diluted in 5 µl Opti-MEM (Invitrogen) was pipetted in 384-well tissue culture plates (Greiner). 0.2 µl Oligofectamine (Invitrogen) was diluted with 4.8 µl Opti-MEM, incubated for 5 min and pipetted to each well of the plate. The mixtures were incubated for 20 min to allow complex formation and 1,000 cells in 40 µl medium were added to each well. Twenty-four hour post-transfection cisplatin (100 ng/ml) or MMC (100 ng/ml) were added for 1 h or cells were exposed to 10 Gy IR. Cells were washed carefully with PBS and new medium was added. After additional 48 h cells were fixed with −20°C cold methanol for 20 min, washed twice with PBS, and blocked with Blocking Buffer (0.2% Gelatin from cold water fish skin (Sigma-Aldrich Chemie) in PBS) for 5 min. Cell nuclei were stained with DAPI (1 µg/ml) and cells were preserved with 0.02% sodium azide in PBS. Images were acquired on an Olympus IX81 microscope (Olympus) and cell numbers were determined using the Scan∧R Analysis software (Olympus). Every knockdown was repeated 3 times. Cell numbers with and without DNA damaging agents were compared to Rluc transfections.

### GammaH2AX Assay

HeLa cells were treated with 10 Gy IR 48 h post esiRNA transfection and fixed 1 h or 6 h later. Cells were stained with a phospho-H2AX antibody (clone JBW301, Upstate Biotechnology, 1∶600 dilution) and with donkey anti-mouse TxRed conjugated antibody (Molecular Probes, 1∶400 dilution). DNA was stained with DAPI (1 µg/ml). Cells were preserved with 0.02% sodium azide in PBS and images were acquired on an Olympus IX81 microscope and analyzed by Scan∧R Analysis software (Olympus). Every knockdown was repeated 3 times. Percentages of gammaH2AX positive cells were compared to Rluc transfections. *p* values were calculated by Student's *t* test.

### Structural Bioinformatics Methods

Sequence-based analysis (Blast) failed to identify any statistically significant sequence homology between KIAA0415 and any previously characterized protein. Fold recognition techniques were applied to search for potential structural homologies of KIAA0415 with known protein structures. The threading algorithm ProHit (ProCeryon Biosciences) was used to search for structural resemblance of the uncharacterized KIAA0415 sequence with protein structures of the Brookhaven Protein Databank (PDB). Threading calculations were performed with parameters and scoring functions as previously published [55]. A fold library consisting of 19.961 protein chains representing the PDB at 95% sequence identity was used. Three-dimensional (3D) models for KIAA0415 were generated by threading its sequence through each fold of the fold library. Inspection of fold coverage, gaps position and content in the sequence-to-structure alignments obtained, together with the analysis of the secondary structure prediction obtained for KIAA0415 by PredictProtein (http://www.predictprotein.org/) were used to discard possible false positives in top scoring folds. A three-dimensional model of KIAA0415 was built based on the threading alignments obtained with high confidence predicted folds and four template structures (PDBId: 2d7d, 2p6r, 1gm5, and 2eyq) by using Modeler in Discovery Studio (Accelrys v1.7). Manual docking of ADP and Mg^2+^ onto the resulting KIAA0415 3D model was done based on the X-ray structures of 2d7d and 1gm5. Refinement of the obtained complex was done with AMBER 10 [56]. A first step of energy-minimization by 1,000 cycles of steepest descent and 500 cycles of conjugate gradient with harmonic force restraints on protein atoms was followed by 3,000 cycles of steepest descent and 3,000 cycles of conjugate gradient without constraints. The system was then heated from 0 to 300K for 10 ps. An equilibration step of 30 ps at 300K was followed by a 10 ns MD productive run. The ff03 force field, periodic boundary conditions at constant pressure with Langevin temperature coupling and Berendsen pressure coupling, TIP3P explicit solvent, counterions, 8 Å cut-off for non-bonded interactions, and the SHAKE algorithm for hydrogens were used.

### BAC Transgeneomics

BAC recombineering and the generation of BAC-transgenic cell lines was performed as described previously [44],[57]. A list of all BACs and primers used in this study is provided in Table S6.

### Immunoprecipitation and Mass Spectrometry Analysis

A goat anti-GFP antibody (MPI-CBG Antibody Facility) immobilized on G-protein sepharose (GE Healthcare) or GFP-Trap (Chromotek) were used for immunoprecipitation [44],[52]. Glycine eluated KIAA0415-LAP and SPG11-LAP complexes were analyzed on silver stained SDS PAGE. Excised slices were in-gel digested and analyzed by nanoLC-MS/MS on a LTQ (Thermo Fisher Scientific) as previously reported [58],[59]. Glycine eluates from KIAA0415-LAP, KIAA0415-NFLAP, SPG11-LAP, and DKFZp761E198-LAP immunopurifications were used for in-solution digestion and analyzed by shotgun-LC-MS/MS on a LTQ Orbitrap (Thermo Fisher Scientific) [60]. Proteins identified in more than 15% of 193 independent immunoprecipitations performed in ongoing collaborations projects from unrelated baits were considered common backgrounds and further excluded.

### Cell Fractionation

Cell fractionation was performed with the commercially available ProteoExtract kit (Novagene, Merck Biosciences) according to the manufacturer's protocol.

### Patients Material

We selected 166 unrelated index cases with spastic paraplegia diagnosed according to the Harding's criteria [61]; 109 had a pure form of the disease and 57 had a complex form partially overlapping with the SPG11 typical phenotype. They included 64 index patients from families with dominant inheritance (mean age at onset: 27.0±16.6 y), 38 index patients with inheritance compatible with an autosomal recessive trait (mean age at onset: 25.6±19.9 y), and 64 patients with no family history of the disease (mean age at onset: 31.2±16.9 y). Most patients were French (*n* = 137) while the remaining patients originated from other countries in Europe (*n* = 16), North-Africa (*n* = 8), or elsewhere (*n* = 5).

This study was approved by the local Bioethics committee (approval No. 03-12-07 of the Comité Consultatif pour la Protection des Personnes et la Recherche Biomédicale Paris-Necker to Drs A. Durr and A. Brice). Informed and written consents were signed by all participating members of the families before blood samples were collected for DNA extraction. All clinical evaluations were performed according to a protocol established by the European and Mediterranean network for spinocerebellar degenerations (SPATAX, coordinator: Dr. A. Durr) that included: a full medical history and examination, estimation of the age at onset by the patient, observation of additional neurological signs, electroneuromyographic (ENMG) studies, and brain MRI, when possible. Disability was assessed on a 7-point scale as previously described [62],[63].

Mutations in SPAST, SPG3, SPG6, and SPG42 were previously excluded in most of the index patients with dominant transmission by direct sequencing and multiplex ligation-dependent probe amplification in the case of SPAST and SPG3 [64] and unpublished data. Among autosomal recessive and sporadic patients, mutations in the CYP7B1/SPG5 gene were excluded in most patients [63] while SPG11 and SPG15 mutations have been excluded in all complex autosomal recessive forms [50].

### Mutation Detection

All coding exons of the gene KIAA0415 (Ensembl gene ID: ENSG00000164917) and its splice junctions were amplified by PCR on a Thermocycler 9700 (Applied Biosystems, Foster City, CA) using specific primers (see Table S7). 3.1 Kb on the 3′ and 1.5 Kb on the 5′-UTRs were also sequenced in patients with an autosomal recessive transmission carrying a single heterozygote variant. The amplicons were sequenced in both directions using the BIGDYE V3 chemistry in an ABI3730 automated sequencer (Applied Biosystems) as recommended by the supplier. The seqscape v2.6 (Applied Biosystems) software was used to highlight nucleotide variations in comparison to the normal consensus sequence of both genes. In family FSP70, the mutation was confirmed after subcloning of PCR products into the pcDNA3.1/V5-His TOPO TA vector using TOP10 bacteria according to the manufacturer's recommendations (Invitrogen) and direct sequencing of at least 5 independent clones of both alleles.

After identification of a variant, reamplification and resequencing was systematically performed. Segregation of the mutations/polymorphisms with the disease was verified by direct sequencing in additional family members whose DNA samples were available. In addition, 79 and 121 unrelated healthy Caucasian and North-African subjects were screened to evaluate the frequency of new nucleotide changes. In order to estimate evolutionary conservation, gene sequences of different species were downloaded from the Ensembl genome browser (www.ensembl.org) and aligned using the ClustalW algorithm (http://www.ebi.ac.uk/Tools/clustalw2/index.html). All variants were systematically tested for their effect on splicing at: http://rulai.cshl.edu/cgi-bin/tools/ESE3/esefinder.cgi, http://rulai.cshl.edu/new_alt_exon_db2/HTML/score.html, http://www.fruitfly.org/seq_tools/splice.html. Predicted effects of missense changes were tested using SIFT and POLYPHEN at http://sift.jcvi.org/www/SIFT_seq_submit2.html and http://genetics.bwh.harvard.edu/pph/.

### Lymphoblast Cell Lines

Cell lines were obtained from patients by infection with Epstein-Barr-Virus (Table S8). Lymphoblast were cultured in RPMI medium supplemented with 1% Pen/Strep, 2 mM L-Glutamine, 10 mM Hepes, 1% Fungizone, and 20% FCS. 200.000 cells were plated in 6-well plates and cultured without or with 10 ng/ml MMC or exposed to 10 ug/ml bleomycin for 1 h. Growth of the cells was monitored daily by counting the trypan blue negative cells using a Countess Automated Cell Counter (Invitrogen). Four days after incubation 100.000 cells were stained with the FITC Annexin V Appoptosis Kit II (BD Biosciences) followed by FACS (BD Biosciences) analyses following the manufacturer's protocol. Experiments were performed two times in duplicates.

## Supporting Information

Figure S1 **3D model of the KIAA0415 SF2 domain (a) and plot of RMSD along the MD simulation (b).**
(3.81 MB EPS)

Figure S2 **KIAA0415 knockdown does not influence I-SceI expression levels.**
(0.69 MB EPS)

Figure S3 **Interacting proteins of the core KIAA0415 protein complex.**
(0.89 MB EPS)

Table S1 **Primary RNAi screening data. Red, esiRNAs that decreased the frequency of homologous recombination below an average z-score of −2.** Green, esiRNAs that increased the frequency of homologous recombination above an average z-score of 2. n.a., not available.
(3.90 MB XLS)

Table S2 **Threading results.**
(0.02 MB XLS)

Table S3 **Proteins identified by nanoLC-MS/MS and by shotgun-LC-MS/MS after immunoprecipitations.** Proteins were considered confident hits when identified with at least three peptides with MASCOT ions score above 20.
(0.06 MB DOC)

Table S4 **List of variants and mutations found in KIAA0415.**
(0.03 MB XLS)

Table S5 **Primer sequences used to generate secondary esiRNAs.** AR, autosomal recessive; AD, autosomal dominant; spo, sporadic cases; ESE, Exonic splicing enhancers; na, not applicable, dbSNP, single nucleotide polymorphisms database at http://www.ncbi.nlm.nih.gov/projects/SNP/.
(0.04 MB XLS)

Table S6 **BAC clones and primer sequences used for BAC tagging.**
(0.02 MB XLS)

Table S7 **List of primers used for KIAA0415 mutation detection.**
(0.02 MB XLS)

Table S8 **Lymphoblast cell lines used for sensitivity experiments.**
(0.01 MB DOC)

Video S1 **Molecular dynamics simulation of the putative helicase C domain in KIAA0415 (see Online Methods).** The protein is shown in a cartoon representation. The SF2 helicase motif regions are shown in colours: I in white, Ia in yellow, II in orange, III in red, IV in cyan, and V in blue. ADP and three residues (E379, D481, and E652) coordinating the Mg^2+^ are shown in sticks and coloured by atom type. Mg^2+^ is represented by a green sphere.
(1.56 MB MPG)

## Abbreviations

BAC: bacterial artificial chromosome
BER: base excision repair
PDB: Brookhaven Protein Databank
DSB: double strand break
HSP: hereditary spastic paraplegia
IR: ionizing radiation
LOH: loss of heterozygosity
MMC: mitomycin C
NER: nucleotide excision repair
NHEJ: nonhomologous end joining
PFA: paraformaldehyde
Rluc: renilla luciferase
TCC: thin corpus callosum
